# Rice (*Oryza sativa* L.) cultivated in the Central Highlands of Vietnam: Dataset on the endophytic microbiome

**DOI:** 10.1016/j.dib.2023.109551

**Published:** 2023-09-07

**Authors:** Dinh Minh Tran, Thi Huyen Nguyen

**Affiliations:** Institute of Biotechnology and Environment, Tay Nguyen University, Buon Ma Thuot, Dak Lak 630000, Vietnam

**Keywords:** Rice endophytic microbiome, Proteobacteria, Gammaproteobacteria, biosynthesis

## Abstract

Rice (*Oryza sativa* L.) is the main annual crop cultivated in the Central Highlands region of Vietnam. Understanding the endophytic bacterial community of this plant, a new technique for sustainable production can be developed. In this work, a representative sample was obtained by combining rice (RVT variety) root samples collected from five different fields in Dray Sap Commune, Krong Ana District, Dak Lak Province, the Central Highlands of Vietnam. Using the Illumina MiSeq technology, the 16S rRNA metagenomics was applied to the sequencing amplicons library. The QIIME2 matched with the SILVA SSURef reference database was employed to analyze the taxonomic profile, and the PICRUSt2 and MetaCyc databases were used to predict the functional profile of rice endophytic prokaryotes. Results revealed that Enterobacterales was the most predominant class (57.7%) in the bacterial community, and biosynthesis was the primary function of the rice endophytic microbiome (75.95%). Raw sequences obtained in this work are available from the National Center for Biotechnology Information (NCBI) (Bioproject ID: PRJNA994482) and Mendeley Data [1]. Data in this work provide insight into the endophytic microbiome of rice (RVT variety) cultivated in the Central Highlands of Vietnam. These data are valuable for developing a new method for producing locally sustainable rice employing endophytic bacteria. This is the first report on the endophytic microbiome of rice cultivated in this region.

Specifications Table

**Table:** 

Subject	Microbiology: Microbiome
Specific subject area	Metagenomics, Molecular biology, Bioinformatics
Data format	Raw, Filtered, and Analyzed
Type of data	Figures
Data collection	- Collection of the rice roots (RVT variety) - Extraction of the microbial metagenomic DNA - Conduction of metagenomic sequencing - Analysis of the metagenomic sequences
Data source location	• Institution: Institute of Biotechnology and Environment, Tay Nguyen University • Commune/District/Province/Region: Dray Sap/Krong Ana/Dak Lak/The Central Highlands • Country: Vietnam • Latitude and longitude coordinates for collected samples: 12°33′49′′N, 107°58′55′′E; 12°33′58′′N, 107°58′53′′E; 12°34′15′′N, 107°58′55′′E; 12°34′03′′N, 107°58′40′′E; 12°34′13′′N, 107°59′04′′E
Data accessibility	Raw sequences Repository name: NCBI SRA Data identification number: Bioproject ID PRJNA994482 Direct URL to data: https://www.ncbi.nlm.nih.gov/bioproject/994482 Repository name: Mendeley Data Data identification number: doi:10.17632/2d92dzsnzn.1 Direct URL to data: https://data.mendeley.com/datasets/2d92dzsnzn/1

## 1 Value of the Data

• Data provide taxonomic and functional profiles of the endophytic microbiome of rice (RVT variety) grown in Vietnam's Central Highlands.
• Data can be helpful for comparing the endophytic microbiome of rice (RVT variety) grown in this region and others.
• Data can be useful for developing a new method for producing locally sustainable rice (RVT variety) employing endophytic bacteria.

One of the top exporters and producers of rice worldwide is Vietnam. The Central Highlands region is the capital of both coffee and black pepper production in the country. Other than coffee and black pepper plants, rice is the main annual crop grown in this region. According to a report, Vietnam had 7,238,900 hectares of rice planted and produced 43,852,600 tons in 2021, in which the Central Highlands contributed 250,200 hectares and 1,466,300 tons, respectively [2]. Farmers frequently use chemical fertilizers to boost the yield of rice products in this area. However, chemical fertilizers can contaminate groundwater, are more ecologically resilient, and reduce soil fertility and microorganisms [3]. To produce rice sustainably, it is believed that employing beneficial microbes is the best option. Rhizospheric and endophytic microbiome data of coffee, black pepper, and sugarcane plants have been presented in order to build a new technique for the sustainable production of the primary crops in this region [4], [5], [6], [7]; nevertheless, the microbiome data of rice is still unknown. This work aimed to establish a dataset of endophytic bacteria of rice (RVT variety) grown in the Central Highlands of Vietnam, using the 16S rRNA metagenomics.

## 2 Data Description

**Fig. 1:**
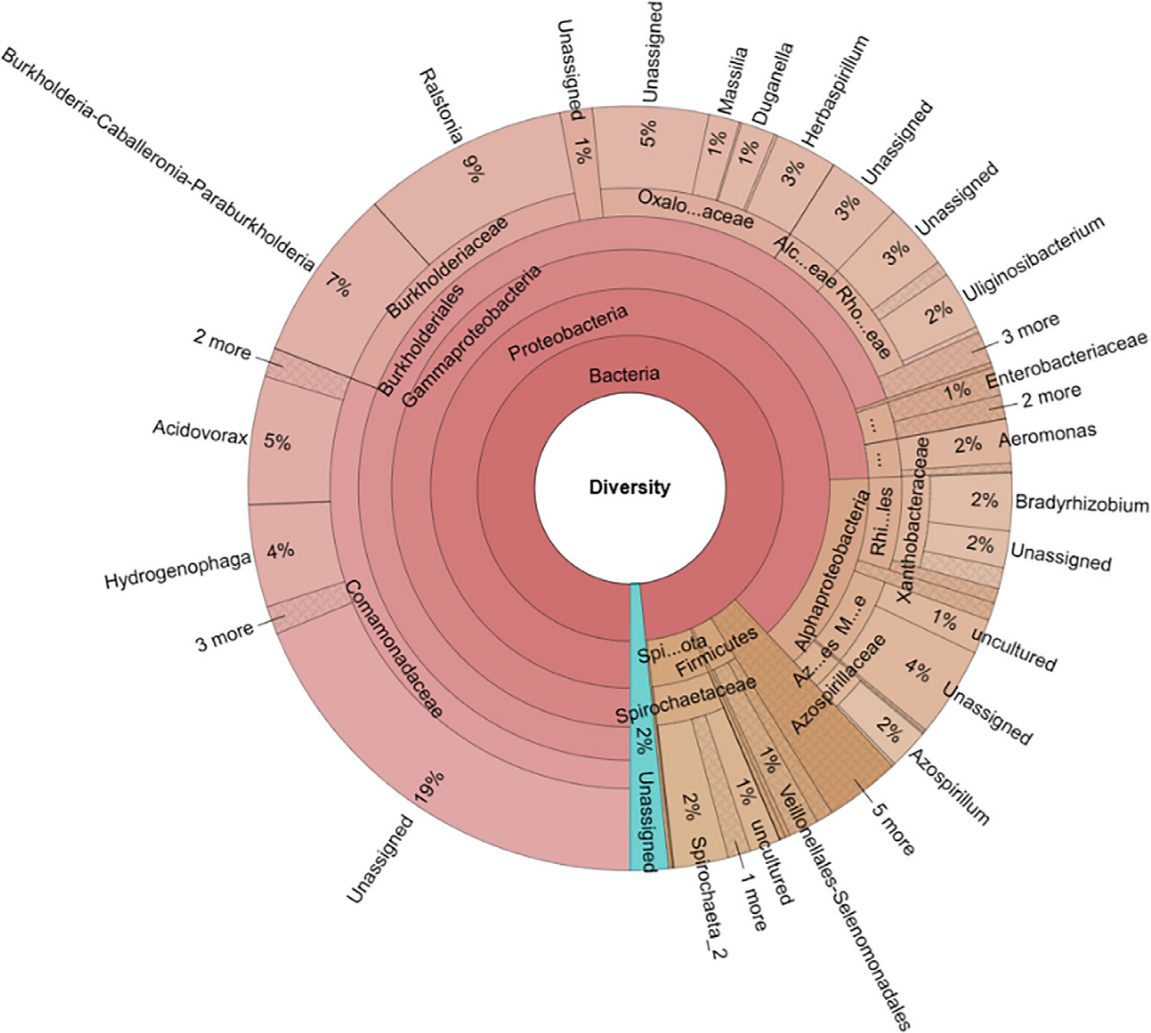
Taxonomic profiles of the endophytic microbiome of rice cultivated in the Central Highlands, Vietnam.

In this work, a total of 174,687 reads was used for data analysis after quality filtering of 232,642 raw reads. The result of the taxonomic analysis (Fig. 1) showed that 13 phyla of bacteria were identified from the root sample of rice. Among them, Proteobacteria (89.39%) was shown to be the major phylum, followed by Spirochaetota (4.75%) and Firmicutes (2.23%). From these phyla, 28 classes were determined. Among these classes, Gammaproteobacteria accounted for 75.52%, Alphaproteobacteria for 13.87%, Spirochaetia for 4.68%, and Negativicutes for 1.31%. A total of 53 orders were found within the recognized classes; among them, Burkholderiales was the most abundant (70.79%), followed by Rhizobiales (5.64%), Micropepsales (5.38%), Spirochaetales (4.68%), Aeromonadales (2.26%), Enterobacterales (2.23%), and Azospirillales (1.95%). Additionally, from the orders, 67 families were detected. Comamonadaceae (31.48%) was the most abundant among these families, followed by Burkholderiaceae (16.34%), Oxalobacteraceae (10.7%), Rhodocyclaceae (6.29%), Micropepsaceae (5.38%), Xanthobacteraceae (5.02%), Spirochaetaceae (4.68%), and Alcaligenaceae (3.22%). Finally, 97 genera were assigned, and Ralstonia dominated with 8.86% of the total genera. The raw data of this work were deposited in the NCBI (can be downloaded at https://trace.ncbi.nlm.nih.gov/Traces/index.html?view=run_browser&acc=SRR25258148&display=download) and Mendeley Data (can be downloaded at https://data.mendeley.com/datasets/2d92dzsnzn/1).

**Fig. 2:**
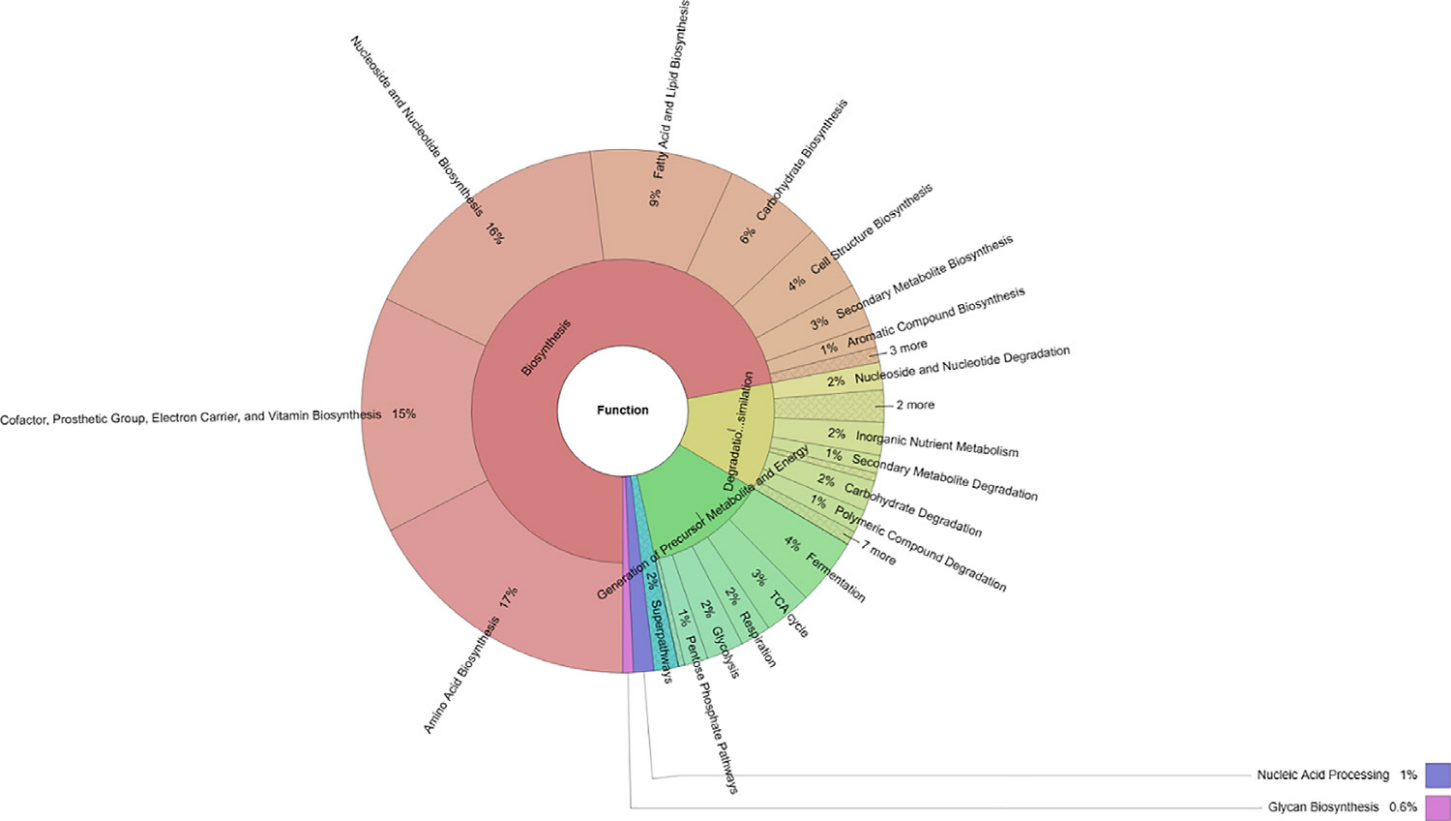
Functional profiles of the endophytic microbiome of rice cultivated in the Central Highlands, Vietnam.

Functional analysis (Fig. 2) revealed that biosynthesis (72%) was the predominant function of the rice endophytic microbiome, followed by the generation of precursor metabolites and energy (13.06%), and degradation/utilization/assimilation (11.5%). Of the functions involved in biosynthesis, amino acid biosynthesis (17.61%) was the most abundant, followed by nucleoside and nucleotide biosynthesis (15.95%); cofactor, prosthetic group, electron carrier, and vitamin biosynthesis (14.59%); fatty acid and lipid biosynthesis (8.89%); carbohydrate biosynthesis (6.05%); cell structure biosynthesis (4.11%); and secondary metabolite biosynthesis (2.65%). The raw data of this work were deposited in the NCBI (can be downloaded at https://trace.ncbi.nlm.nih.gov/Traces/index.html?view=run_browser&acc=SRR25258148&display=download) and Mendeley Data (can be downloaded at https://data.mendeley.com/datasets/2d92dzsnzn/1).

## 3 Experimental Design, Materials and Methods

### 3.1 Sampling

On October 30, 2021, five samples of rice roots of RVT variety (each weighing 50–70 g) were taken from five different farms in Dray Sap Commune, Krong Ana District, Dak Lak Province. The roots were collected from 5 distinct locations in each field and then combined into one sample. Finally, the five samples were mixed well and combined into one representative sample. The sample was stored in an ice box (4°C) and transferred to the laboratory within 2 h. In the laboratory, the root surface was sterilized as previously described [7] to remove microorganisms that adhere to the root surface. The sterilized sample was then maintained at −80°C until analysis.

### 3.2 Genomic DNA Extraction, Library Preparation, and Sequencing

Following the manufacturer's instructions, 300 mg of the root sample was used to extract the metagenomic DNA of the rice endophytic microbiome using the DNeasy PowerSoil Pro kit (Qiagen, Germany). The 16S rRNA genes of the extracted metagenomic DNA were amplified, and libraries of the amplicons were then created using the Swift amplicon 16S plus internal transcribed spacer panel kit (Swift Biosciences, USA). Finally, the amplicons from the library were sequenced using the Illumina MiSeq platform (2 × 150 PE) [8].

### 3.3 Bioinformatic Analysis of Data

Data analysis using bioinformatics was done, as previously mentioned [8]. Briefly, Bcl2fastq was used to demultiplex raw basecall sequences. Raw basecall sequences were demultiplexed using Bcl2fastq. Adapters, primers, and low-quality sequences (average score of <20 and read length of <100 bp) were removed using the Trimmomatic 0.39 and Cutadapt 2.10. Clustering and dereplication of reads into amplicon sequence variants were carried out using the q2-dada2 plugin and QIIME2 pipeline 2020.8. Taxonomic analysis of rice endophytic microbiome was performed using QIIME2, which was aligned with the SILVA SSURef reference database. The functional profile of rice endophytic prokaryotes was predicted using the PICRUSt2 2.3.0-b and MetaCyc databases.

## Limitations

Not applicable.

## Ethics Statement

The current work does not involve human subjects, animal experiments, or any data collected from social media platforms.

## CRediT authorship contribution statement

**Dinh Minh Tran:** Conceptualization, Methodology, Investigation, Formal analysis, Software, Data curation, Validation, Visualization, Writing – original draft, Writing – review & editing. **Thi Huyen Nguyen:** Investigation, Formal analysis.

## Data Availability

Root endophytic microbiome dataset of rice (Oryza sativa L.) grown in the Central Highlands of Vietnam (Original data). Root endophytic microbiome dataset of rice (Oryza sativa L.) grown in the Central Highlands of Vietnam (Original data).
